# Rapid Detection of *Prunus Necrotic Ringspot Virus* by Reverse Transcription-cross-priming Amplification Coupled with Nucleic Acid Test Strip Cassette

**DOI:** 10.1038/s41598-017-16536-6

**Published:** 2017-11-23

**Authors:** Ya-Yun Huo, Gui-Fen Li, Yan-Hong Qiu, Wei-Min Li, Yong-Jiang Zhang

**Affiliations:** 1 Biotechnology Research Institute, Chinese Academy of Agricultural Sciences, Beijing, 100081 China; 2 Chinese Academy of Inspection and Quarantine, Beijing, 100176 China

## Abstract

*Prunus necrotic ringspot* virus (PNRSV) is one of the most devastating viruses to *Prunus* spp. In this study, we developed a diagnostic system RT-CPA-NATSC, wherein reverse transcription**-**cross**-**priming amplification (RT-CPA) is coupled with nucleic acid test strip cassette (NATSC), a vertical flow (VF) visualization, for PNRSV detection. The RT-CPA-NATSC assay targets the encoding gene of the PNRSV coat protein with a limit of detection of 72 copies per reaction and no cross-reaction with the known *Prunus* pathogenic viruses and viroids, demonstrating high sensitivity and specificity. The reaction is performed on 60 °C and can be completed less than 90 min with the prepared template RNA. Field sample test confirmed the reliability of RT-CPA-NATSC, indicating the potential application of this simple and rapid detection method in routine test of PNRSV.

## Introduction

*Prunus necrotic ringspot virus* (PNRSV), a member of genus *Ilarvirus*, is one of the most devastating pathogens in *Prunus* spp. In addition to the cultivated *Prunus* species such as apple (*Malus pumila*), plum (*P. domestrica*), almond (*P.armeniaca*), sweet cherry (*P. avium*) and peach (*P. persica*), this virus also naturally infects hop (*Humulus lupulus*) and rose (*Rosa spp*.)^1^. PNRSV usually is transmitted by plant propagation materials including seedling, pollen, scion, stock and seed^2–4^, thus making the virus easily spread among countries and regions in particular through international trade. Since the first identification of PNRSV in 1941 in the United States^5^, the virus so far has been recorded in more than 40 nations and regions of Europe, Asia, Africa, South America, North America and Oceania^6^. PNRSV engenders representative necrotic spots, random shot-holes, and sometimes mottling, rugosity and distortion in young leaves of *Prunus* spp. hosts, and the PNRSV-infected fruits grow slowly with delayed ripening, finally causing low fruit production and significant economic losses^7–10^.

Regarding the huge threat to the stone fruit production, PNRSV is listed as a quarantine plant virus in P. R. China. With the aim to prevent the virus spread, several methodologies, including enzyme-linked immunosorbent assay (ELISA) and molecular-based techniques such as reverse transcription-polymerase chain reaction (RT-PCR), Real-time fluorescence RT-PCR and RT-loop-mediated isothermal amplification (LAMP), have been employed for PNRSV detection. However, ELISA detection of PNRSV often presents the false negative results because one specific immune serum can hardly recognize all PNRSV isolates^11,12^, and in particular the PNRSV titer in the dormant branch is usually below the detection limit of ELISA. *S Spiegal* used ELISA and RT-PCR to detect same bark samples collected from dormant PNRSV-infected trees. The result shows only two of seventeen samples tested positive by ELISA, whereas RT-PCR showed all samples but one produced a virus-specific band^13^. While RT-PCR and RT-LAMP are sensitive enough to detect PNRSV in dormant branch compared with ELISA^14,15^, the former requires running gels which is time-consuming as well as increase the risk of contamination, and the latter has relative low specificity due to the use of staining dyes for color development of the amplicon^16^. Real-time fluorescence RT-PCR is a rapid detection method for PNRSV but requires the expensive equipment and reagents^17^. The limitations and defects of these detection methods do affect their application in routine diagnosis of PNRSV.

Cross-priming amplification (CPA) is a recently developed isothermal DNA amplification method with high specificity and sensitivity^18^ and has been successfully used to detect number of human, animal and plant pathogens ranging from bacteria to virus^16,19–23^. Nucleic acid test strip cassette (NATSC) bases on the vertical flow hybridization and is designed to visually detect double-labeled amplicons in only 5 to 10 min^24^. In this study, we established a method termed RT-CPA-NATSC, in which, CPA was combined with reverse transcription (RT) to amplify the RNAs of PNRSV, and the resulting amplicons were subsequently visualized with NATSC. The specificity and sensitivity of RT-CPA-NATSC assay in detecting PNRSV were evaluated, demonstrating that this method has great potential for PNRSV surveillance.

## Results

### Screening of primer set for RT-CPA detection of PNRSV

**Figure 1:**
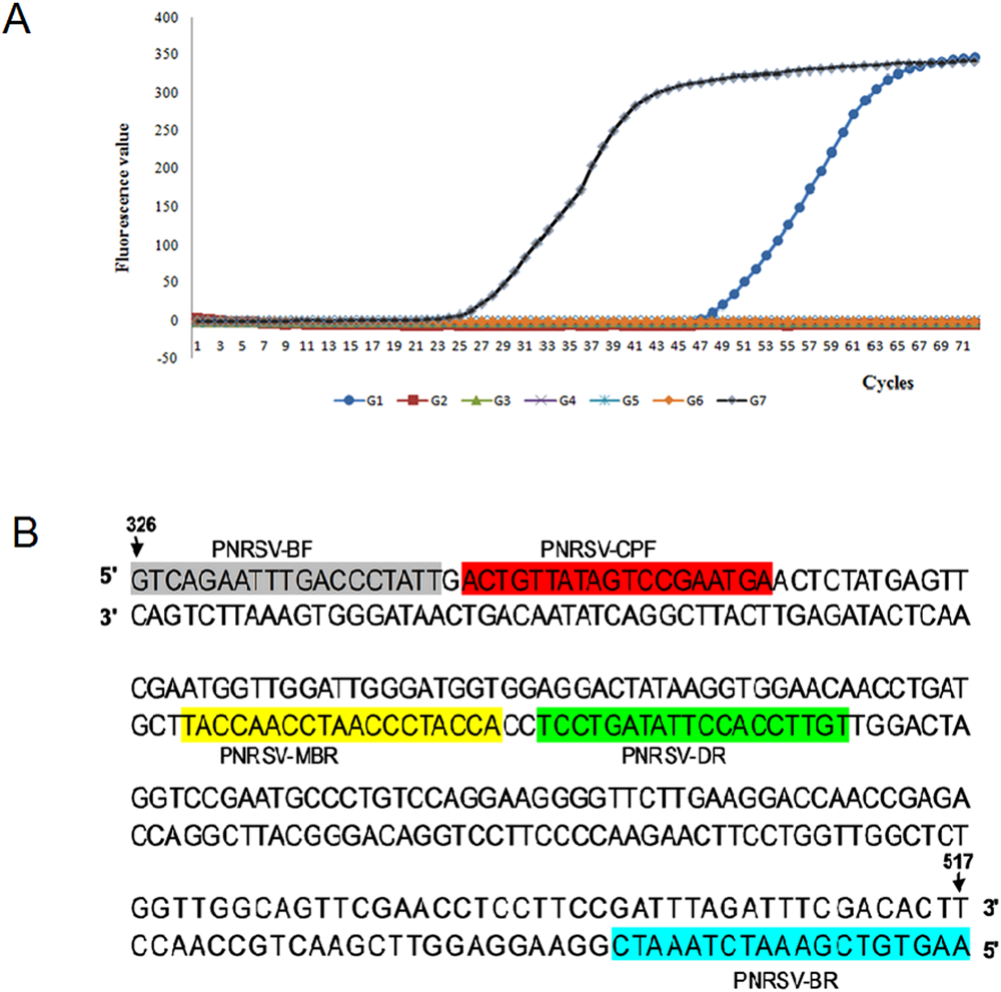
Primer set screening for RT-CPA assay of PNRSV. (**A**) Reaction curves of real-time RT-CPA. Fluorescence was monitored every 60 s, and one cycle shown here represents 1 min. (**B**) Target sequence and primer location of the primer set G7. Nucleotide positions are based on the PNRSV coat protein (CP)-encoding gene (Genbank No: NC-004364.1). The cross primer (5′-TGTTCCACCTTATAGTCCTGTACTGTTATAGTCCGAATGA-3′) covers the PNRSV-CPF (5′ to 3′) and PNRSV-MBR (3′ to 5′) sequences.

By using total RNA extracted from the PNRSV-infected cucumber (*Cucumis sativus*) leaves as template, a total of seven primer sets (Supplementary Table 1) were evaluated on their reaction efficiency for PNRSV detection by RT-CPA coupled with real-time fluorescence at 60 °C. As shown in Fig. 1A, few fluorescent signals were detected from reactions with the primer sets G2, G3, G4, G5 and G6, whereas both G1 and G7 gave strong signals. However, in view of their amplification curves, the primer set G7 (Fig. 1B) demonstrated better efficiency than that of the primer set G1, and was selected to perform the RT-CPA-NATSC assay hereinafter.

### Development of RT-CPA-NATSC for PNRSV detection

**Figure 2:**
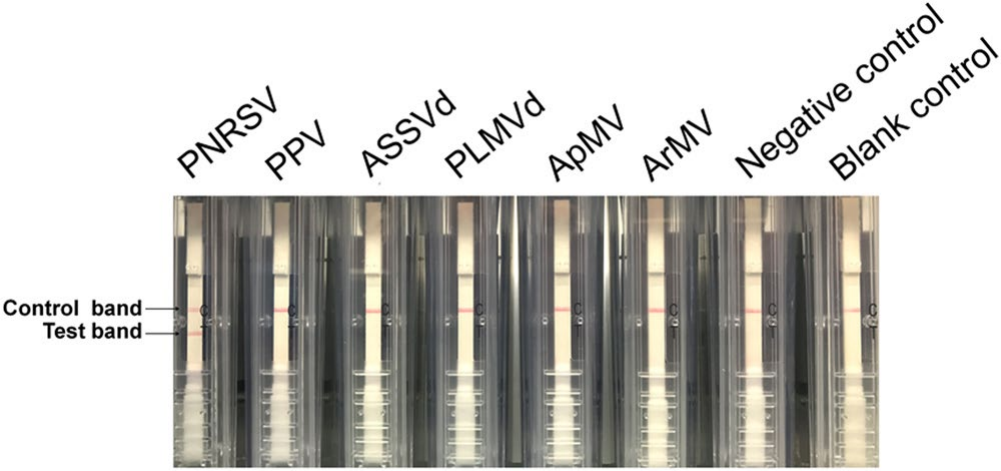
Specificity of RT-CPA-NATSC on the PNRSV detection. The reaction on PNRSV (lane 1) clearly showed both the test line and the control line, but only the control line were observed from the five common *Prunus* spp. pathogens, PPV, ASSVd, PLMVd, ApMV and ArMV (lane 2–6), as well as negative control and blank control (lane 7 and lane 8). Negative control, Totol RNA of healthy cucumber leaves; Blank control, RNase-free distilled water.

With the selected primer set G7, RT-CPA was performed on total RNA of the PNRSV-infected cucumber leaves with 40 min, following visualization of the resulting amplicons with NATSC. As expected, both the test and control line positions turn red within 5 to 10 min (Fig. 2, lane 1). In contrast, the negative reactions based on the total RNA from healthy cucumber leaves as well as RNase-free distilled water showed only in the control line (Fig. 2, lane 7,8).

### Specificity evaluation of RT-CPA-NATSC on the PNRSV detection

Five common *Prunus* pathogenic viruses and viroids, *Plum pox virus* (PPV), *Apple mosaic virus* (ApMV), *Arabis mosaic virus* (ArMV), *Apple scar skin viroid* (ASSVd), and *Peach latent mosaic viroid* (PLMVd), were included herein to assess the specificity of RT-CPA-NATSC on the PNRSV detection. As shown in Fig. 2, only the reaction on PNRSV present one red line at the position of the test line, making a clear differentiation between PNRSV and the other tested *Prunus* pathogens, and indicating a 100% analytical specificity of RT-CPA-NATSC on PNRSV.

### Sensitivity evaluation of RT-CPA-NATSC on the PNRSV detection

**Figure 3:**
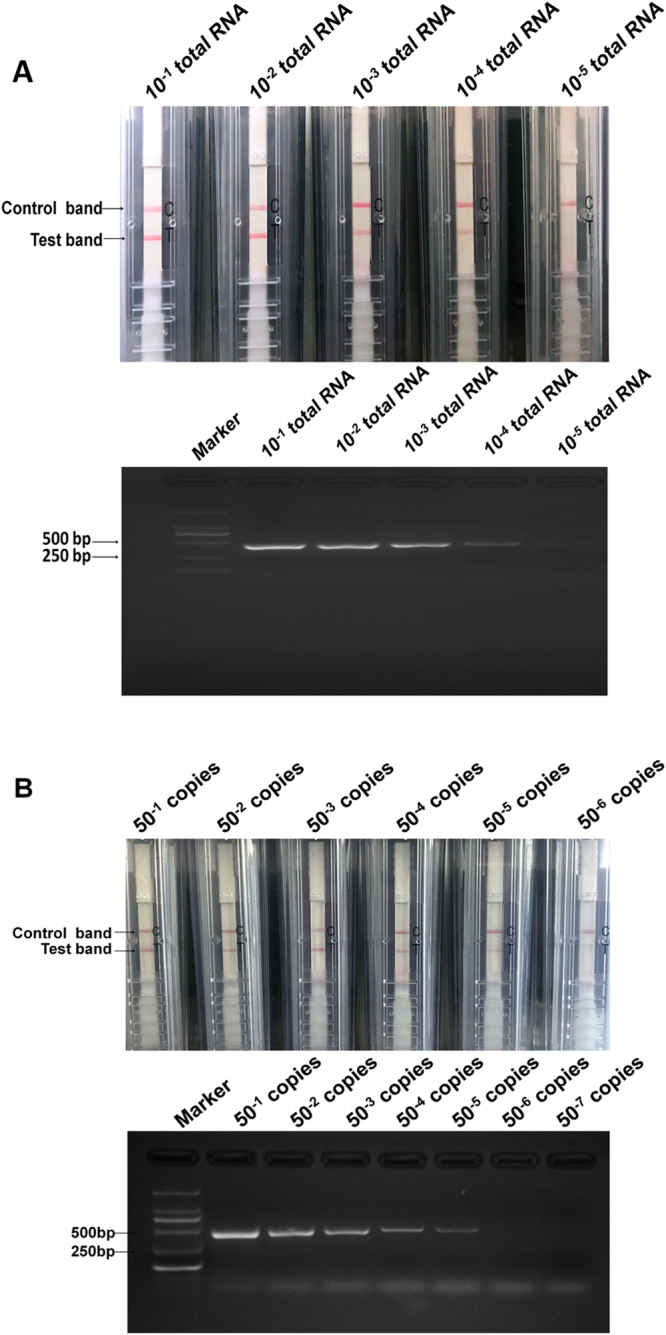
Sensitivity of RT-CPA-NATSC on PNRSV detection. (**A**) Comparison of the sensitivities of RT-CPA-NATSC (the upper pannel) and RT-PCR (the lower pannel) on the PNRSV detection. Lane1–5 represent 10^−1^–10^−5^ dilution of total RNA of the PNRSV-infected cucumber sample, respectively. (**B**) The detection limit of RT-CPA-NATSC on PNRSV. Lane 1–6 represent 50^−1^–50^−5^ dilution of pPNRSV-CP, respectively. Both the test line and the control line were indicated in lane 1-5.

A serial 10-fold dilutions of total RNA (48 ng/μL) prepared from the PNRSV-infected cucumber leaves were used as templates to define the sensitivity of RT-CPA-NATSC on the PNRSV detection. As a result, the reactions on 10^−4^ diluted RNAs consistently showed both the control line and the test line, while no test line was observed from the 10^−5^ diluted RNA samples, indicating a similar threshold of RT-CPA-NATSC (uppenal of Fig. 3A) to that of RT-PCR on PNRSV detection (lowerpenal of Fig. 3A), about total RNA 4.8 × 10^−3^ ng/μL.

The limit of detection (LOD) of RT-CPA-NATSC on PNRSV was further determined by testing serial 50-fold dilutions of a plasmid pPNRSV-CP (58 ng/μL) which bear a 455 bp DNA fragment corresponding to the PNRSV CP gene. With the molecular weight of the plasmid and insert know as well as the concentration of pPNRSV-CP plasmid measured by micro-spectrophotometer and the copies was calculated using the equation below^25^.

Copies/ul = 6.02 × 10^23^(copy/mol) × DNA amount ng/ul × 10^−9^)/(DNA length(bp) × 660(g/mol/bp)

The reactions remain positive up to the 50^−5^ dilution (Fig. 3B), showing that the LOD of RT-CPA-NATSC is of 72 copies of pPNRSV-CP (upperpenal of Fig. 3B), which has similar sensitivity of 72 copies with RT-PCR RT-PCR (lowerpenal of Fig. 3B).

### Validation of RT-CPA-NATSC on PNRSV detection with field samples

**Figure 4:**
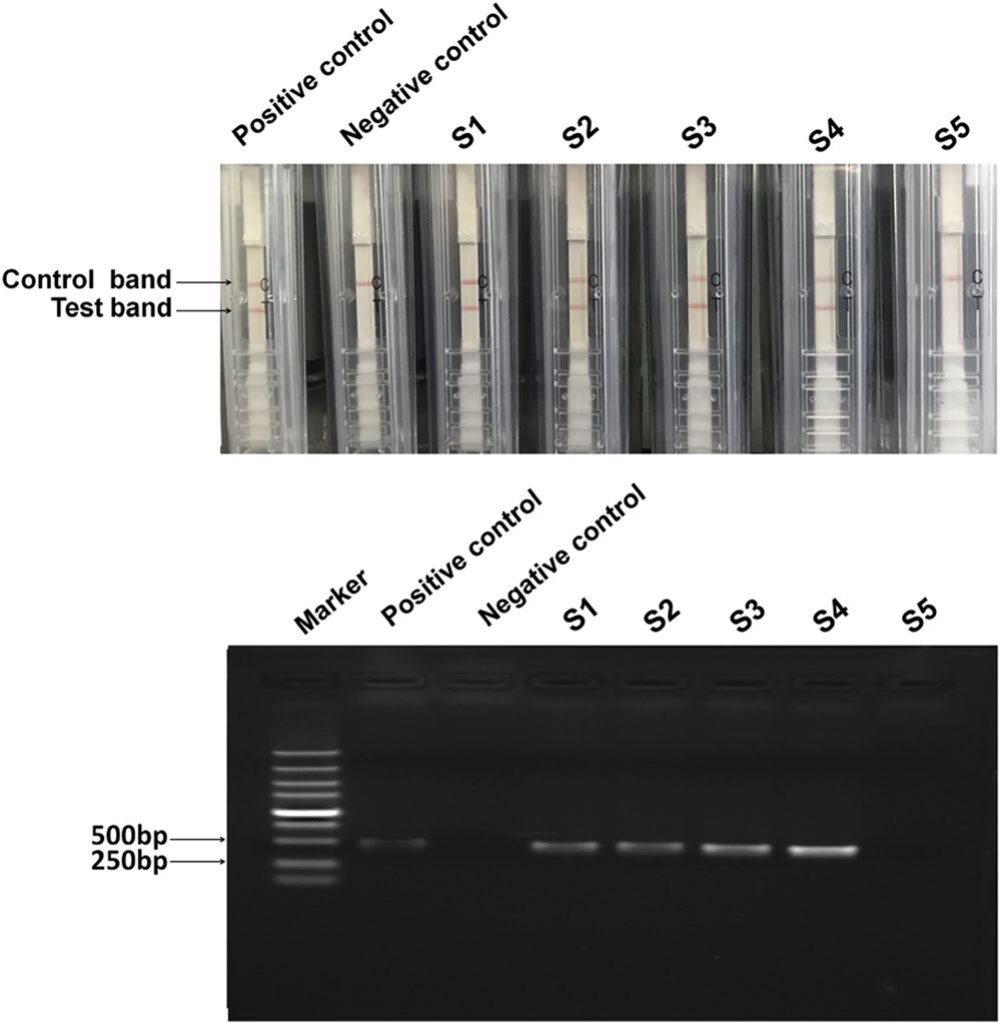
Detection of PNRSV in field samples with RT-CPA-NATSC (the upper pannel) and RT-PCR (the lower panel). Positive control, Total RNA of PNRSV infected cucumber leaves; Negative control, Total RNA of healthy cherry leaves; S1-S5, Total RNA of samples 1-5.

A total of five cheery plant leaf samples were tested by using RT-CPA-NATSC. Four samples (S1, S2, S3 and S4) were positive with the exception of S5, consistent with the results derived from RT-PCR (Fig. 4) and double antibody-sandwich ELISA (DAS-ELISA) (Table 1), indicating the reliability of RT-CPA-NATSC to detect PNRSV in field samples.

**Table 1:** DAS-ELISA detection of PNRSV in field samples.

Sample	Positive control	Negative control	S1	S2	S3	S4	S5
OD_405_*	0.943	0.074	0.302	0.255	0.291	0.861	0.091

^*^If the OD_405_ value of the sample/ that of negative control were ≥ 2, the sample is recognized as a positive sample.

## Discussion

The method of CPA coupled with nucleic acid test strip has been successfully applied in the *in vitro* diagnosis of viruses of human and animal^16,19–23^. However, there are few reports about its application in the detection of plant viruses. In the current study, a high specific and sensitive diagnostic system RT-CPA-NATSC, wherein RT-CPA is combined with NATSC, is developed to detect PNRSV, an important pathogen causing severe yield losses on stone fruit worldwide^2^.

In agreement with the specificity and sensitivity of RT-CPA-NATSC on detection of red-spotted grouper nervous necrosis virus^16^, Porcine Epidemic Diarrhea Virus^22^, and Thrombocytopenia Syndrome Virus^23^, RT-CPA-NATSC on the PNRSV detection showed 100% analytical specificity as well as the similar sensitivity with that of RT-PCR, thus exhibiting potential application on diagnosis of the low titer of PNRSV within the dormant branch^13^. However, compared with RT-PCR, qRT-PCR as well as ELISA, this diagnostic system requires only a simple heat block or water bath but not expensive or large equipments. In particular, due to coupling with NATSC, the resulting amplicons of RT-CPA can be visualized on a lateral flow strip housed in an enclosed, sealed plastic device with no more than 10 min, circumventing the time-consuming process of gel electrophoresis and stain as well as eliminating the false positives probably caused by environmental contamination. Field samples tested by using RT-CPA-NATSC demonstrated a 100% agreement with RT-PCR and DAS-ELISA. In *X Zong*’s research, the detectability of RT-LAMP is 19.6 × 2^−6^ ng/μL total RNA and 19.6 × 2^−4^ ng/μL total RNA of RT-PCR whereas the limit of RT-CPA-NATSC is 48 × 10^−4^ ng/μL total RNA, which indicate RT-CPA-NATSC is a high sensitivity and specificity of this method on the PNRSV detection^14^.

As one of the commonly used isothermal amplification detection methods, LAMP is known to have a higher sensitivity compared with RT-PCR^13^. Visual inspection of the LAMP amplification products by turbidity can indicate the presence of amplified DNA, however, the weakly positive samples are usually hard to be distinguished from the negative samples with naked eyes. In contrast, the RT-CPA-NATSC has more advantages to recognize the weakly positive results and the negative results through visualizing the appearance of the test line and the control line.

In conclusion, a method RT-CPA-NATSC with high specificity and sensitivity was developed herein for the PNRSV detection. Due to the advantages of visualization, easy manipulation and time-saving, this method is eligible for application in elementary inspection and quarantine departments in PNRSV routine test, and provided a reference to detect other plant viruses.

## Materials and Methods

### Virus and viroid samples and total RNA extraction

The virus and viroid standard samples were collected from different sources (Table 2). Of them, PNRSV, PPV, ArMV were individually propagated in *Cucumis sativus, Nicotiana benthamiana* and *N. tabacum cv* White Burley. In addition, ASSVd and ApMV were propagated in apple (*M. ylvestris*), and PLMVd is in peach (*P. persica*). The field cheery samples were collected from five cheery trees which had suspected symptoms of necrotic ringspot in Tai mountain (Shandong Province, China) in 2010, August and stored at Chinese Academy of Inspection and Quarantine at the temperature of −80 °C. Total RNA was extracted by using the Plant Total RNA Extraction Kit (Tiangen, China) following the manufacturer’s protocols and was measured with ND-1000 Spectrophotometer (NanoDrop Technologies). Notably, all the healthy plants used for virus and viroid propagation were tested by RT-CPA-NATSC assay. As results, only control band appeared on the nucleic acid test strip, whereas no test band was visualized. Thus, the healthy cucumber leaves were selected as negative control (NC) in RT-CPA-NATSC assay, and RNase-free distilled water (dH_2_O) were used as blank control (BC).

**Table 2:** Viruses and viroids used for the developmention of RT-CPA-NATSC assay.

Sample	Provider
*Prunus necrotic ringspot virus* (PNRSV)	American Type Culture Collection (Manassas, VA, USA), ATCC (PV-552)
*Arabis mosaic virus* (ArMV)	American Type Culture Collection (Manassas, VA, USA), ATCC (PV-192)
*Plum pox virus* (PPV)	Deutsche Sammlung von Mikroorganismen und Zellkulturen (Braunschweig, German), DSMZ (PV-0001)
*Apple scar skin viroid* (ASSVd)	Chinese Academy of Agricultural Sciences (Beijing, China)
*Peach latent mosaic viroid* (PLMVd)	Beijing Entry-Exit Inspection and Quarantine Bureau (Beijing, China)
*Apple mosaic virus* (ApMV)	China Agricultural University (Beijing, China)

### Primers design

A primer pairs was designed to amplify the total RNA extracted from infected leaf tissue by RT-PCR. based on the capsid protein gene of PNRSV. The sequence of forward primer: 5′- GGT CCC ACT CAG GGC TCA AC-3′, reverse primer: 5′- CGC AAA AGT GTC GAA ATCTAA ATC -3′. The amplification product was cloned into plasmid pPNRSV-CP. A set of primers was designed with software Oligo 6.0(Molecular Biology Insights, West Cascade, CO, U.S.A.). The five primer sequences are demonstrated in Supplementary Table 1.

### Real-time fluorescent PCR

Real-time fluorescent PCR was used to select the best primer from seven sets on the Roche LightCycler® 480 II system (Germany). The reaction volume is of 20 μL including 1 μL RNA template (48 ng/μL), 1 μL PNRSV-CPR (20 μM), 0.6 μL PNRSV-MBF (20 μM), 0.6 μL PNRSV-DF (20 μM), 0.2 μL PNRSV-BF (20 μM), PNRSV-BR (20 μM), 0.8 μL dNTP (10 mM), 0.6 μL MgSO_4_ (100 mM), 1 μL Bst DNA polymerase (8 U/μL), 1 μL AMV reverse transcription polymerase (10 U/μL), 0.5 μL Betaine (5 mol/L), 2 μL 10× Thermopol buffer, 0.5 μL SYTO® 9 Green Fluorescent Nucleic Acid Stain (100 μM) (Invitrogen). Reaction was carried out at a isothermal temperature of 60 °C for 90 min.

### RT-CPA-NATSC assay

Seven RT-CPA-NATSC assay primer sets were designed based on the PNRSV *CP* gene (NC_004364.1) using software Oligo 6.0 (Molecular Biology Insights, West Cascade, CO, USA). Each set contains five primers (supplementary file 1): BF and BR were outer primers, MBF/MBR and DF/DR were inner primers with label of Biotin and 6-FAM, respectively, and CPF/CPR was cross-primer. The RT-CPA was first performed using the same reaction mixture as Real-time PCR assay but without SYTO® 9 Green Fluorescent Nucleic Acid Stain and reactions were conducted at a constant temperature of 60 °C for 90 min. A drop of paraffin oil was dropped on the reaction mixture to keep it from contamination. After amplification, the tube was put into nucleic acid strip detection cassette (Ustar, Hangzhou, China) and detection result was directly observed through visual inspection. Both test and control bands can be observed in the positive reactions, whereas the negative reactions show only the control band.

### RT-PCR assay

The PNRSV specific RT-PCR primers PNRSV-F and PNRSV-R (Supplementary Table 1) were designed based on the PNRSV *CP* gene (NC_004364.1). RT-PCR was performed using PrimeScript^TM^ One-Step RT-PCR Kit (Takara, Dalian, China) in a final volume of 50 μL including 2 μL PrimeScript 1 Step Enzyme Mix, 25 μL 2 × 1 Step Buffer, 1 μL PNRSV-F (10 μM), 1 μL PNRSV-R (10 μM), 1 μL template RNA and 20 μL RNase Free dH_2_O. Reactions were run as follows: 50 °C for 30 min; 94 °C for 2 min; 30 cycles of 94 °C for 30 sec, 58 °C for 30 sec, and 72 °C for 40 sec. The PCR amplification products were then examined by agarose gel electrophoresis (1.5% agarose, TAE), followed by ethidium bromide staining.

### DAS-ELISA

Field samples were tested for PNRSV with DAS-ELISA kit (Agdia Inc., Elkhart, IN, USA) following the manufacture’s procedure. Healthy cherry leaves were individually used as the negative control. The OD values of DAS-ELISA plates were recorded at 405 nm using an Thermo Multiskan Ascent. Once the OD value of sample/ that of negative control is ≥ 2, the sample is deemed as a positive sample.

## Electronic supplementary material

Supplementary table 1
