# Supplementary material for: Rapid Detection of Prunus Necrotic Ringspot Virus by Reverse Transcription-cross-priming Amplification Coupled with Nucleic Acid Test Strip Cassette
Source: Sci Rep. 2017 Nov 23;7:16175. doi: 10.1038/s41598-017-16536-6 (PMC5700948; doi:10.1038/s41598-017-16536-6)
Supplement: Supplementary file 1 — Supplementary table 1 [file 41598_2017_16536_MOESM1_ESM.pdf]

Title of manuscript: Rapid Detection of Prunus Necrotic Ringspot Virus by Reverse Transcription-cross-priming Amplification Coupled with Nucleic Acid Test Strip Cassette

Author list: Ya-Yun Huo, Gui-Fen Li, Yan-Hong Qiu, Wei-Min Li, Yong-Jiang Zhang

Supplementary table 1. Oligonucleotide of primers sets used for RT-PCR and RT-CPA-NATSC assay of PNRSV.

Sets	Name	Sequence (5′-3′)	length(mer)
RT-PCR	PNRSV-F	GGTCCCACTCAGGGCTCAAC	20
	PNRSV-R	CGCAAAAGTGTCGAAATCTAAATC	24
G1	PNRSV-BF	CGACAGAGGCAGTGAAGTA	19
	PNRSV-CPF	GTTCATTCGGACTATAACAGCTCACGACCACTCTCCCTCA	40
	PNRSV-DR	FMA-CAATAGGGTCAAATTCTGA	19
	PNRSV-MBR	Bio-GTTCATTCGGACTATAACA	19
	PNRSV-BR	CATCCCAATCCAACCATTC	19
G2	PNRSV-BF	CACTCTCCCTCAGTTGATG	19
	PNRSV-MBF	Bio-CCGTGAGAGGTCCGAATGT	19
	PNRSV-DF	FMA-GGGTTTGTAGCACACAATC	19
	PNRSV-CPR	CGTGAAGTCTATACTCAAGTACCGTGAGAGGTCCGAATGT	40
	PNRSV-BR	CATCAACTGAGGGAGAGTG	19
G3	PNRSV-BF	TTGTAGCACACAATCACCG	19
	PNRSV-MBF	Bio-GTGACGACGACAGAGGCAG	19
	PNRSV-DF	FMA-CTTGAGTATAGACTTCACG	19
	PNRSV-CPR	CATCAACTGAGGGAGAGTGCAGTGACGACGACAGAGGCAG	40
	PNRSV-BR	CAATAGGGTCAAATTCTGA	19
G4	PNRSV-BF	CTTGAGTATAGACTTCACG	19
	PNRSV-MBF	Bio-CCACTCTCCCTCAGTTGAT	19
	PNRSV-DF	FMA-GTCAGAATTTGACCCTATT	19
	PNRSV-CPR	ATCCCAATCCAACCATTCGATCCACTCTCCCTCAGTTGAT	40
	PNRSV-BR	CCACCTTATAGTCCTCCAC	19
G5	PNRSV-BF	GTCCGAATGAACTCTATGA	19
	PNRSV-CPF	TCGGTTGGTCCTTCAAGAATGCGAATGGTTGGATTGGGAT	40
	PNRSV-DR	FAM-GTTCCACCTTATAGTCCTC	19
	PNRSV-MBR	Bio-TCGGTTGGTCCTTCAAGAA	19
	PNRSV-BR	AAAGTGTCGAAATCTAAAT	19
G6	PNRSV-BF	AGCACACAATCACCGAGAG	19
	PNRSV-CPF	CATCAACTGAGGGAGAGTGCTACGACAGAGGCAGTGAAGT	40
	PNRSV-DR	FAM-TCGTGAAGTCTATACTCAA	19
	PNRSV-MBR	Bio-CATCAACTGAGGGAGAGTG	19
	PNRSV-BR	CAATAGGGTCAAATTCTGA	19
G7	PNRSV-BF	GTCAGAATTTGACCCTATT	19
	PNRSV-CPF	TGTTCCACCTTATAGTCCTGTACTGTTATAGTCCGAATGA	40
	PNRSV-DR	FAM-ACCATCCCAATCCAACCAT	19
	PNRSV-MBR	Bio-TGTTCCACCTTATAGTCCT	19
	PNRSV-BR	AAGTGTCGAAATCTAAATC	19
